# Health equity requires transformational change: Financial incentives based on worn-out market thinking will not deliver

**DOI:** 10.1371/journal.pgph.0003147

**Published:** 2024-04-25

**Authors:** Els Torreele, Heather Sherwin

**Affiliations:** 1 Independent Researcher and Advisor, Geneva, Switzerland; 2 Director Impact Investing, ELMA Philanthropies Services, Cape Town, South Africa; PLOS: Public Library of Science, UNITED STATES; McGill University, CANADA

The Covid-19 vaccine market concentration and hoarding that left many Global South countries unable to access vaccines in a timely manner have led to calls to expand and decentralize manufacturing capacity as a critical element towards more equitable access to vaccines globally [1–3]. Multiple initiatives are underway to build manufacturing infrastructure in low- and middle income countries (LMICs). However, without proper attention to who owns and controls the production and underlying technologies, there is a risk that well-meaning donor investments reinforce market dynamics that favour a handful of major international producers over truly local efforts [4]. This is particularly relevant for the African Vaccine Manufacturing Accelerator (AVMA), the new US$ 1Bn financing instrument approved by the Board of Gavi, the Vaccine Alliance, in December 2023 [5].

Three AVMA design issues are cause of concern. First, current wording in AVMA documents defines local production as ‘”geographically located on the African continent” [5]. Any international company that sets up a subsidiary in Africa and produces vaccines, which in newspeak is referred to as ‘localized’ production, is eligible for the new financing instrument. However, *localized* production is fundamentally different from *local* production in which actual ownership, management and strategic decision-making are rooted in the local (national or regional) socio-economic ecosystem and respond to local health needs, strategic objectives, and/or market opportunities. There is no reason to assume that localized production by global companies would result in more equitable outcomes than what we saw during the pandemic. Johnson & Johnson (J&J)’s production in South Africa of Covid-19 vaccines that were exported to Europe while South Africa was unable to access any vaccines is a case in point [6].

To deliver health equity and security, production must be truly locally governed and as described above, embedded in national or regional health and economic development policy. Without political support and targeted financial incentives designed to address their specific needs, [7] African vaccine manufacturers will not be able to compete with established international companies that already have capabilities, knowhow and a strong financial base.

Second, AVMA’s financial incentives are structured to foster localization efforts by international producers, to the detriment of truly local efforts. The first financial award provided by AVMA is a milestone payment (pull incentive) upon achieving WHO Prequalification [5]. This means that only companies with access to at-risk capital to finance product development and building a regulatory dossier can achieve this high bar.

Established vaccine producers have the capital and can benefit from government or philanthropic grant funding for product research and development (R&D) up to registration, especially in areas considered market failures [8]. In contrast, emerging African companies with production capability struggle to attract appropriate financing for bringing pharmaceuticals to market, with only hard currency debt from development finance institutions (DFIs) available. DFIs are currently unable or unwilling to finance product development, nor are they providing grant funding which would be the most appropriate (push) incentive to support such efforts, including the incremental innovation and optimization needed to adapt originator products for use in resource-poor and community settings.

For instance the South African company Afrigen, despite being at the heart of a WHO-coordinated effort to decentralize mRNA manufacturing capacity has major challenges to attract grant funding to build the mRNA product pipeline that is essential for the long term sustainability of the initiative (*Terblanche P*., *personal communication*) [9]. Similarly, Biovac in South Africa struggles to raise the roughly US$15 million needed to take an oral cholera vaccine through bio-equivalence studies and regulatory approval; the Institut Pasteur Dakar requires US$20–30 million for clinical trials of its measles rubella vaccine. Because such vaccines must be available at the lowest possible price to enable equitable access, profit margins will be minimal. Clinical development and building a regulatory dossier can therefore not be funded through expensive debt or equity financing, which would require future profits to pay back or share dividends with capital providers.

**If AVMA wants to support local producers, filling this gap in push financing should be a critical niche to focus on, instead of the current preference for pull incentives**.

Thirdly, AVMA’s design assumes that local producers must (eventually) compete in the global vaccine market: its second financial incentive is a price subsidy when winning competitive Gavi-UNICEF tenders (another pull incentive) [5]. This success criterion is based on the mistaken belief that market competition is an effective way to deliver timely and equitable access to health technologies [10]. It also overlooks that current vaccine market dynamics are far from a level playing field, with unequal market powers resulting from layers of historical technological, legal and regulatory monopolies. In 2022 and excluding Covid-19, four international companies (MSD, Pfizer, GSK, Sanofi) captured 73% of the global vaccine market by value, producing 24% of the doses by volume [11]. In contrast, the Serum Institute of India produced 20% of all vaccine supplies, representing only 2% of the global market value.

This deeply entrenched dual-market situation reflects the challenges LMIC manufacturers face to move beyond large-scale manufacturing at low-cost and invest in innovative capacities and/or focus on smaller-scale markets and diversification, even if that would better address local needs. A narrow focus on competitiveness of individual companies or products in a cut-throat market with unequal market powers also fails to acknowledge the many socio-economic and health multipliers of local production and economic ecosystem development. These include job creation, skills development, local technological innovation, local component supply chain opportunities and increased pharmaceutical supply security and resilience to ensure more timely and equitable access.

The belief in competition as driver for health security is at the heart of AVMA’s financial incentives: companies must first compete to achieve WHO-PQ, including to attract investments from international capital markets, and subsequently they compete in international tenders to obtain an order. Such winner-takes-it-all market power dynamics, which tend to favour large international and Global North producers and economies-of-scale, are reminiscent of neoliberal economic policies, but should not be guiding global health efforts to increase health equity in 2024. They also wrongly assume that capital is readily available in African markets: Africa focused funds represented 0.1% of the funds closed in 2023, i.e. US$1,07Bn versus US$784.73Bn globally [12].

Some argue that it is likely faster and more cost-effective for donors to support well-established large-scale producers. However such considerations ignore the legitimate strategic aspiration of countries and regions for greater self-determination, self-reliance and health security, including through autonomous technological capacity for critical health supplies as is being spearheaded by countries like Brazil through health-industrial policy [13]. They also overvalue efficiency as the core objective of economics over other societal goals like social justice, health resilience or equity, an approach increasingly questioned by economists themselves [14].

Learning lessons from Covid-19, now is the time to build a conducive R&D and manufacturing ecosystem in the Global South, in which truly local companies have agency and are supported politically and financially to address priority health needs, fostering collective intelligence and collaboration over competition [15]. As new programmes such as AVMA and EU’s Global Gateway are designed, it is critical they target the needs of emerging local producers, which include access to affordable capital to finance at-risk the technical work needed to adapt, optimize, and establish a regulatory dossier for submission to regulatory authorities and other push incentives. Business-as-usual market dynamics will not deliver equity.

## References

[1] Africa CDC. Partnerships for African Vaccine Manufacturing (PAVM) Framework for Action [Internet]. [cited 2023 Oct 9]. Available from: https://africacdc.org/download/partnerships-for-african-vaccine-manufacturing-pavm-framework-for-action/

[2] European Commission. Global Gateway: EU steps up support for global health and equitable access to health products and local manufacturing [Internet]. [cited 2024 Jan 28]. Available from: https://ec.europa.eu/commission/presscorner/detail/en/ip_23_5278

[3] Dzau VJ, Balatbat CA, Offodile AC. Closing the global vaccine equity gap: equitably distributed manufacturing. Lancet. 2022;399(10339):1924–6. doi: 10.1016/S0140-6736(22)00793-0 35533706 PMC9075857

[4] Torreele E. Tackling vaccine inequity in 2023: have we made progress? Expert Review of Vaccines. 2024;23(1):1–4. doi: 10.1080/14760584.2023.2292771 38078804

[5] Gavi, the Vaccine Alliance. The African Vaccine Manufacturing Accelerator: what is it and why is it important? [Internet]. [cited 2024 Jan 28]. Available from: https://www.gavi.org/vaccineswork/african-vaccine-manufacturing-accelerator-what-and-why-important

[6] Robbins R, Mueller B. Covid Vaccines Produced in Africa Are Being Exported to Europe. The New York Times [Internet]. 2021 Aug 16 [cited 2023 Oct 3]; Available from: https://www.nytimes.com/2021/08/16/business/johnson-johnson-vaccine-africa-exported-europe.html

[7] Greenslade L, Sherwin H. Devex. 2024 [cited 2024 Mar 1]. Opinion: Will new global health spending deliver major health gains? Available from: https://www.devex.com/news/sponsored/opinion-will-new-global-health-spending-deliver-major-health-gains-107073

[8] Lalani HS, Nagar S, Sarpatwari A, Barenie RE, Avorn J, Rome BN, et al. US public investment in development of mRNA covid-19 vaccines: retrospective cohort study. BMJ. 2023 Mar 1;380:e073747. doi: 10.1136/bmj-2022-073747 36858453 PMC9975718

[9] Medicines Patent Pool. Global mRNA Technology Transfer Programme [Internet]. 2022 [cited 2024 Jan 29]. Available from: https://medicinespatentpool.org/what-we-do/mrna-technology-transfer-programme

[10] Torreele E, McNab C, Adeyi O, Bonnell R, Dhaliwal M, Hassan F, et al. It is time for ambitious, transformational change to the epidemic countermeasures ecosystem. The Lancet. 2023 Mar 25;401(10381):978–82. 10.1016/S0140-6736(23)00526-3 36924776

[11] WHO Global Vaccine Market Report 2022. [Internet]. 2022 [cited 2023 Oct 4]. Available from: https://www.who.int/publications/m/item/global-vaccine-market-report-2022

[12] Lau K, Lemonius D. Private Equity International’s Fundraising Report FY2023 [Internet]. 2024. Available from: https://media.privateequityinternational.com/uploads/2024/02/2023-full-year-fundraising-report-pei.pdf

[13] Urias E. The potential synergies between industrial and health policies for access to medicines: insights from the Brazilian policy of universal access to HIV/AIDS treatment. Innovation and Development. 2019 Jul 3;9(2):245–60.

[14] Deaton A. Rethinking Economics or Rethinking My Economics [Internet]. IMF. [cited 2024 Mar 19]. Available from: https://www.imf.org/en/Publications/fandd/issues/2024/03/Symposium-Rethinking-Economics-Angus-Deaton

[15] Torreele E, Wolfe D, Kazatchkine M, Sall A, Ruxrungtham K, Fitchett JRA, et al. From private incentives to public health need: rethinking research and development for pandemic preparedness. The Lancet Global Health. 2023 Oct 1;11(10):e1658–66. doi: 10.1016/S2214-109X(23)00328-5 37652070

